# Pathways of *Escherichia coli* transfer from animal manure: risks and mitigation in agriculture

**DOI:** 10.3389/fpubh.2025.1568621

**Published:** 2025-06-06

**Authors:** Nunzio Sarnino, Subhasish Basak, Lucie Collineau, Roswitha Merle

**Affiliations:** ^1^Veterinary Centre for Resistance Research Berlin, Institute of Veterinary Epidemiology and Biostatistics, Freie Universität Berlin, Berlin, Germany; ^2^University of Lyon–French Agency for Food, Environmental and Occupational Health and Safety (ANSES), Epidemiology and Surveillance Support Unit, Lyon, France

**Keywords:** *Escherichia coli*, environmental contamination, animal manure, crops contamination, water contamination, soil contamination

## Abstract

Animal manure is applied in agriculture to improve soil fertility and crop yield. Nonetheless, manure can also carry *Escherichia coli (E. coli)*, including antibiotic-resistant strains. Therefore, it may pose a risk for environmental contamination. This review includes 50 studies which were identified from the search terms related to the transmission of *E. coli* through manure. The review outlines the potential routes of *E. coli* transmission from manure to soil, water and crops and which factors most critically determine persistence and contamination. The persistence of *E. coli* in soil is highly variable, ranging from <30 days for composted manures to more than 200 days in cooler conditions. These differences depend on the type of manure used, the environmental conditions and the treatment employed. While crops can be contaminated directly through application of manure, contaminated irrigation water may be a more important pathway. The foremost cause of surface water contamination seems to be rainfall runoff, whereas groundwater contamination is rather uncommon, mainly happening in areas with specific soil conditions. Composting and adherence to pre-harvest intervals are very effective mitigation strategies that can greatly reduce contamination risks. Overall, this review identifies research gaps on water contamination pathways and the persistence of resistant strains. Moreover, it sets up the basis for the development of robust risk assessments and evidence-informed approaches to address the contamination risks that are linked to animal manure.

## Introduction

While animal manure is an effective fertilizer that enhances soil fertility and crop yields (1) it can serve as a source of microbial contamination, including *Escherichia coli* (*E. coli*). These bacteria may migrate from animal manure to the environment, as for example into soil (2), water (3), and crops (4, 5), thereby posing potential risks for environmental contamination. Furthermore, once established in the environment, they may affect human health through different pathways, such as fresh vegetable consumption (6), recreational swimming (7) or direct contact with grazing animals (8). Organic fertilizers derived from livestock manure can also contain various antimicrobial resistance genes (ARGs) (9, 10), which may be introduced into soil and water systems (11). To create effective mitigation strategies, it is essential to identify major contamination pathways and understand how *E. coli* survives in various environmental settings.

One of the main goals of the ENVIRE project (http://www.envire-project.de) is investigating how broiler chicken manure might facilitate the dissemination of resistant *E. coli* into the environment, ultimately affecting human health. To address these challenges, the project adopts various on-farm interventions and manure treatments. In parallel, the project implements a Quantitative Microbial Risk Assessment (QMRA) model to evaluate human exposure from different pathways and the effectiveness of several interventions (12).

While various bacteria can indicate fecal contamination, this review focuses solely on *Escherichia coli*. That choice reflects the ENVIRE project's mandate and the need to harmonize with our project partners. We excluded Polymerase chain reaction (PCR)-only ARG studies, because our QMRA model requires counts of *E. coli* colony-forming-units (CFUs) rather than gene presence alone, since no dose-response relationship exists for genes.

This review aims to (i) identify non-negligible pathways for the transfer of *E. coli* from animal manure to the environment, relevant to the QMRA; (ii) compile existing quantitative data (e.g., survival and concentration of *E. coli*) across these pathways to inform the QMRA; (iii) outline effective interventions that mitigate manure-derived *E. coli* contamination; and (iv) pinpoint research gaps where further data are needed. By mapping these pathways, the review seeks to strengthen the evidence base for subsequent risk modeling and guide policy and practical interventions to minimize contamination risks.

## Materials and methods

### Literature search strategy

A literature search was conducted using PubMed and Google Scholar. Search terms included combinations of the following keywords:

- “*E. coli*” OR “*Escherichia coli*”
- “antibiotic-resistant *E. coli*”
- “manure”
- “soil contamination”
- “water contamination”
- “agricultural crops”
- “environmental pathways”

The search focused on studies published from 2000 onwards to ensure relevance to contemporary agricultural practices and public health concerns.

### Selection criteria

Studies were included if they satisfied at least one of these criteria:

- Focused on *E. coli* in manure-amended agricultural contexts, highlighting the potential for contamination of soil, water, or crops.
- Provided insights into the persistence, transmission, or mitigation of *E. coli* in agricultural contexts.
- Discussed agricultural management practices relevant to manure application.

Exclusion criteria included studies focused solely on non-agricultural environments or lacking explicit relevance to *E. coli*.

### Data synthesis

Data from the selected studies was collected and synthesized to identify common themes and patterns. The review was organized according to three environmental pathways:

- *Soil contamination*
- *Crops contamination*
- *Water contamination*

Within each pathway, key findings on *E. coli* persistence, factors influencing survival, and potential mitigation measures were extracted.

We applied a qualitative environmental-contamination risk-ranking to summarize how relevant each pathway is for the persistence and transfer of *E. coli* in environmental compartments, based on the strength of evidence found. Each was assigned a risk level: *low, medium*, or *high*. This ranking reflects expert judgement and follows the approach described in FAO/WHO (13). While our primary focus is on environmental contamination, these rankings also inform potential human-exposure risks in downstream assessments.

## Results and discussion

### Description of the studies

In total 50 studies were included in the synopsis. Geographically, 25 of them were from North America, 15 from Europe, four from Asia, three from Africa, two from South America, and one from Oceania. Regarding study type, 27 were field studies, 14 were lab studies, five were modeling studies, and four were reviews (Table 1). The manure investigated in these studies came from cattle, poultry, swine, or horse.

**Table 1:** List of studies included.

**Authors**	**Pathway**	**Region**	**Type of study**
Agga et al. (2024)	Soil contamination	USA	Field study
Alegbeleye et al. (2020)	Water contamination	Brazil	Review
Amato et al. (2020)	Water contamination	USA	Field study
Arnaud et al. (2015)	Soil contamination	Canada	Field study
Atanasova et al. (2025)	Soil contamination	Germany	Lab study
Avery et al. (2004)	Crops contamination	UK	Field study
Black et al. (2021)	Soil contamination	Northern Ireland	Review
Çekiç et al. (2017)	Soil contamination	USA	Lab study
Chapman et al. (2018)	Water contamination	Canada	Modeling
Chuwku et al. (2023)	Soil contamination	Nigeria	Modeling
Cook et al. (2011)	Water contamination	USA	Lab study
Darkazanli and Kiseleva (2019)	Crops contamination	Russia	Lab study
Detert et al. (2021)	Soil contamination	Germany	Lab study
Ekman et al. (2021)	Soil contamination	Australia	Field study
Entry et al. (2004)	Soil contamination	USA	Field study
Fatoba et al. (2022)	Soil contamination	South Africa	Field study
Forslund et al. (2011)	Water contamination	Denmark	Field study
Franz et al. (2008)	Soil contamination	Netherlands	Lab study
Gagliardi and Karns (2000)	Soil contamination	USA	Lab study
Habteselassie et al. (2010)	Crops contamination	USA	Lab study
Holvoet et al. (2013)	Crops contamination	Belgium	Field study
Howard et al. (2016)	Soil contamination	USA	Field study
Hubbard et al. (2020)	Water contamination	USA	Field study
Ingham et al. (2004)	Crops contamination	USA	Field study
Islam et al. (2004)	Crops contamination	USA	Field study
Islam et al. (2005)	Crops contamination	USA	Field study
Iwu and Okoh (2019)	Water contamination	South Africa	Review
Jacobs et al. (2019)	Water contamination	USA	Field study
Jensen et al. (2013)	Crops contamination	Denmark	Field study
Kljujev et al. (2015)	Crops contamination	Serbia	Field study
Marutescu et al. (2022)	Soil contamination	Romania	Review
Merchant et al. (2012)	Soil contamination	Canada	Field study
Mootian et al. (2009)	Crops contamination	USA	Lab study
Mügler et al. (2021)	Water contamination	Laos	Field study
Okada et al. (2024)	Soil contamination	Argentina	Field study
Pang et al. (2020)	Soil contamination	USA	Modeling
Sharma et al. (2019)	Soil contamination	USA	Field study
Sheng et al. (2019)	Soil contamination	USA	Lab study
Siller et al. (2019)	Soil contamination	Germany	Field study
Solomon et al. (2002)	Crops contamination	USA	Modeling
Sowah et al. (2020)	Water contamination	Canada	Field study
Subirats et al. (2021)	Soil contamination	USA	Lab study
Sun et al. (2021)	Crops contamination	Japan	Field study
Suzuki et al. (2024)	Crops contamination	Canada	Field study
Thomas et al. (2024)	Soil contamination	Germany	Field study
Tien et al. (2017)	Water contamination	Netherlands	Modeling
van Overbeek et al. (2021)	Crops contamination	China	Field study
Wang et al. (2021)	Soil contamination	USA	Lab study
Weller et al. (2017)	Soil contamination	China	Lab study
Yao et al. (2013)	Soil contamination	Poland	Lab study

## Soil contamination

### Duration of persistence

This section explores *E. coli* survival in soils amended with both fresh and treated manure, focusing on the role of environmental and management factors (Table 2).

**Table 2:** Studies including data regarding persistence of *E. coli* in agricultural soil.

**Authors**	**Last detection**	**Sampling times**	**Type of manure**	**Composted/fresh**
Avery et al. (2004)	120 days (sheep), 162 days (cattle, pig)	Day 0, 2, 4, 6, 16, 23, 49, 63, 78, and every 14 days until day 218	Cattle, sheep, swine manure	Fresh
Detert et al. (2021)	42 days (22°C), 84 days (4°C)	Days 0, 21, 42, 63, 84	Cattle manure	Fresh
Ekman et al. (2021)	50 days	Days 0, 7, 12, 19, 28, 35, 42, 50	Poultry litter, cow manure	Fresh
Ekman et al. (2021)	Up to 50 (both)	Days 0, 6, 16, 27, 38, 49 (soil); days 42, 49 (lettuce for pathogens).	Poultry litter, cattle manure	Fresh
Entry et al. (2005)	*E. coli* 1 day, enteroccoccae 294 days	Days −1, 1, 7, 14, 28, 179, and 297	Cattle manure	Composted
Fatoba et al. (2022)	42 days	Days 0, 7, 14, 21, and 42	Poultry litter	Fresh
Franz et al. (2008)	54–105 days	6 samplings	Cattle manure	Fresh
Habteselassie et al. (2010)	Up to 41 (end of experiment)	Days 15, 27, 32, 41, and 50	Cattle manure	Fresh
Ingham et al. (2004)	168 days	Biweekly intervals up to 168 days	Cattle manure	Fresh
Islam et al. (2004, 2005)	154 days (alkaline-stabilized dairy manure compost), 196 days (poultry manure compost, dairy manure compost)	Days 0, 7, 14, 21, 35, 42, 49, 70, 84, 91, 105, 112, 126, 140, 154, 168, 182, 196	Poultry litter and cattle manure	Composted
Merchant et al. (2012)	Up to 210 days	August (day 0), September, October, November, December, January, and March	Poultry litter	Fresh
Mootian et al. (2009)	Up to 9 days	Days 3, 6, and 9	Cattle manure	Fresh
Pang et al. (2020)	Modeled survival up to 150 days (dairy manure), up to 120 days (poultry litter), and up to 90 days (horse manure)	Biweekly samplings over 12 trials at varying intervals	Cattle manure, poultry litter, horse manure	Fresh
Sharma et al. (2019)	90 days (poultry litter), 60 days (dairy manure), 45 days (horse manure)	Days 0, 1, 3, 7, 14, 28, 56, 90, 120, and 150	Poultry litter, horse manure, cattle solids	Fresh
Sheng et al. (2019)	60 days	Periodic samplings over 12 trials across three seasons (specific intervals not provided)	Cattle manure	Composted and fresh
Solomon et al. (2002)	Up to 9 days	Days 1, 3, and 5 post-inoculation (lettuce tissues); days 3, 6, and 9 for soil	Cattle manure	Fresh
Subirats et al. (2021)	<30 days	Days 0, 7, 30, and at vegetable harvest	Poultry litter	Composted and fresh
Suzuki et al. (2024)	60 days	Days 0, 7, 60	Cattle manure	Composted
van Overbeek et al. (2021)	272 days	At planting (day 0), 39 days (lettuce), 90 days (leek 2018), 272 days (leek 2019).	Cattle manure	Fresh

### Fresh manure

Field trials have shown that fresh manure often sustains *E. coli* for weeks to months under real-world conditions. Fresh manure may support the extended survival of *E. coli* in soil due to its nutrient-rich composition. Fatoba (14), with *E. coli* persisting up to 42 days in soils amended with fresh chicken manure Likewise, Ekman (16) reported the survival of *E. coli* in soils fertilized with fresh poultry litter and cow manure for up to 50 days in Australia. In another research, *E. coli* persisted in soils amended with cattle, sheep, and swine manure for up to 19 weeks, with swine manure supporting the longest survival (17). A long persistence was also observed by Ingham (18), 168 days in soils treated with non-composted bovine manure, and by van Overbeek (19), up to 272 days in soils treated with fresh cow manure in the rhizosphere of leek crops in the Netherlands. Merchant et al. observed long survival, with *E. coli* detected for up to 210 days. However, a genotype analysis revealed that only a small portion of *E. coli* originated from the manure (20).

Under controlled conditions, lab experiments have detailed how temperature and moisture shape persistence. For example, Habteselassie (15) observed up to 41 days of survival in soils amended with fresh dairy manure. Çekiç et al. (21) demonstrated that environmental conditions strongly influence *E. coli* survival, with significantly longer persistence during cooler fall conditions (up to 280 days) compared to warmer summer conditions, where survival durations ranged from 84 to 112 days depending on the site. Furthermore, in a study by Detert and Schmidt, longer survival was observed at 4°C (84 days) compared to 22°C (42 days) (22). In a research performed in the USA (23), no *E. coli* were detected after 60 days with both raw and composted manure. However, the authors did not take intermediate sampling, making it impossible to determine which type of manure application led to shorter *E. coli* survival. Similarly, Franz et al. (24) observed survival durations of between 54 and 105 days in soil mixed with cattle manure, kept at 16°C in experimental setting.

Pang (25) modeled persistence for up to 90 days under optimal moisture conditions in soils treated with raw manure, pointing up the impact of environmental factors. Poultry litter supported shorter survival durations compared to cow manure, spotlighting the role of manure type. In contrast, in a modeling study by Sharma et al. (26) the longest survival was observed with poultry litter (90 days), followed by dairy manure (60 days), and horse manure (45 days).

### Treated manure

Well-composted manure has been shown to promote microbial degradation processes, further reducing bacterial survival (27).

Several field studies reported shorter *E. coli* survival durations highlighting the effectiveness of composting. For instance, composted cattle manure restricted *E. coli* persistence for up to 60 days in soils under experimental conditions in Japan (28). Even shorter durations, consistently below 30 days, were observed with composted chicken manure, accompanied by significant reductions in *E. coli* populations and associated antibiotic-resistance genes, both in field and laboratory settings (5).

In another field study, fecal coliforms and enterococci persisted for at least 42 weeks in soils amended with dairy manure, while *E. coli* populations dropped below detection levels within a day (29).

From other field studies, it appears that composting primarily impacts *E. coli* survival by reducing initial concentrations rather than significantly shortening persistence durations. For example, *E. coli* O157:H7 persisted for 154 to 217 days in soils treated with poultry or dairy manure composts, with poultry manure generally supporting longer survival, potentially due to its higher nitrogen content (30, 31).

Interestingly, while some studies highlight shorter durations with composted manure, experiments conducted in laboratory report extended persistence, suggesting that factors beyond manure treatment, such as soil composition and environmental conditions, may play a more dominant role (21, 32).

### Manure treatments

Composting is one of the most common treatments for reducing *E. coli* populations in manure. A study by Thomas (33) evaluated different composting configurations, including uncovered static piles, covered static piles, and periodically turned piles. Their results showed that *E. coli* was undetectable within 24 h in all configurations. A key factor was the temperature, that exceeding 50°C, caused the total pathogen inactivation. Proper composting techniques for poultry litter are crucial for reducing antibiotic-resistant *E. coli* (AREc) and associated antibiotic residues. For example, mechanically aerated piles with optimized carbon-to-nitrogen ratios (e.g., C:N = 30) significantly decrease AREc levels, providing dual benefits of improving manure quality and reducing the spread of antimicrobial resistance (AMR) (34).

Similarly, in a model by Tien et al. it was observed that composted dairy manure contained lower levels of antibiotic resistance genes compared to raw or digested manure. According to the study, composting effectively reduces the initial abundance of resistant bacteria and ARGs, although persistence in soil post-application remains dependent on environmental conditions and manure composition (35).

Anaerobic digestion (AD) of chicken manure achieves rapid pathogen inactivation under mesophilic conditions. In experimental trials, total and antibiotic-resistant *E. coli* concentrations fell below detection limits in just 14 days at 30°C and 7 days at 37°C (36). However, Weibull-model simulations indicate that residual *E. coli* cells after AD may persist in soil longer than those from composted manure, suggesting that a brief post-AD composting step would optimize both log-reduction efficacy and environmental decay rates (37).

Broiler litter short-term storage is another practical measure for decreasing *E. coli* concentrations. Extended-spectrum beta-lactamase (ESBL)-producing *E. coli* levels may decline by more than 2 log_10_ within 72 h during summer storage, primarily due to elevated temperatures in deeper litter layers. However, reductions may be less consistent during winter, due to the significant role of environmental conditions. To address this, longer storage periods have been suggested for colder climates to achieve greater reductions (38).

Dairy manure management systems have also evidenced significant efficacy in reducing *E. coli* levels. Howard et al. highlighted that tiered management practices, such as separating solid from liquid waste, were particularly effective, achieving up to a 3-log reduction in *E. coli* concentrations. These systems reduce *E. coli* populations by exposing the bacteria to stress-inducing environments, such as drying beds and lagoons. Advanced separation techniques not only reduce bacterial concentrations but also impact the diversity of *E. coli* populations, limiting the presence of potentially pathogenic strains (39).

Collectively, these findings underscore the complex relation between manure type, treatment status, and environmental conditions in shaping *E. coli* persistence in soil environments. Treated manure primarily serves to reduce contamination risks, though it does not universally limit pathogen survival durations. Persistence levels depend on environmental factors and manure management practices. Proper composting and soil incorporation generally lead to significant bacterial decay, so we assigned those pathways a *low* or *medium environmental contamination risk-ranking*. However, in specific scenarios, such as loamy soils or environments with limited microbial competition, *E. coli* can survive for longer periods (5, 23, 25).

## Crop contamination: is manure application a significant threat?

The extent to which manure application may be a risk for crops contamination depends on multiple factors. It may act as a source of *E. coli* under certain circumstances, though it may not always be the primary vector compared to other crops contamination sources.

Regarding the direct manure-crops contamination, it has been observed that *E. coli* O157:H7 can persist on the surfaces of lettuce and parsley for up to 77 days following the application of raw manure before planting (30). Similarly, *E. coli* were detected on up to 54% of lettuce grown in soils amended with slurry, with splash events during irrigation or rainfall identified as key mechanisms for transferring bacteria to crop surfaces (4). In addition, in experimental settings, it appears that also the lowest bacteria concentration inoculated in manure and water (10^4^ CFU/g) can lead to the colonization of plant surfaces and internal tissues (40). However, the results are heterogenius, and in another experiment *E. coli* persisted in both rhizosphere and bulk soil but was not detected on the lettuce phyllosphere after day 27 post-fresh manure application (15). Similarly, in further experiment, it has been found that *E. coli* can survive in the root zones of lettuce and leek for over 200 days. However, minimal transfer to edible portions suggests that while manure may introduce *E. coli* into the soil, its direct impact on crops is often limited (19). Moreover, it has been demonstrated that applying dairy manure at least 4 months before raspberry harvest results in no detectable *E. coli* on fruits, even when raw manure is used (23).

In a model by Solomon et al. *E. coli* O157:H7 could be transmitted from manure and contaminated irrigation water to lettuce plants, entering through the root system and migrating to internal plant tissues. This internalization makes the bacteria inaccessible to surface sanitizing treatments, such as chlorine rinses, which are typically used to reduce microbial contamination (41). Oppositely, on the field, Ekman et al. (16) observed that, even applying fresh manure, crop contamination was minimal, with no pathogens detected on mature lettuce at harvest.

Similarly, Suzuki et al. (28) detected ARG-bearing coliforms in the root and stem zones, but no *E. coli* was identified on the edible portions of the corn, provided the manure was fully composted. A study by Ingham et al. showed that vegetables grown in soil fertilized with non-composted manure could harbor *E. coli*. While indigenous *E. coli* levels in soil decreased by about 3 log CFU/g within 90 days, low concentrations (0.9–1.6 CFU/g) persisted for over 100 days and were detected up to 168 days post-manure application. Sporadic contamination was observed on washed carrots and lettuce, with occasional positive results even beyond the 120-day harvest interval (18).

However, proper manure management can significantly reduce these risks Additionally, fully composted manure has been shown to eliminate detectable *E. coli* on corn crops, effectively mitigating contamination risks (28).

Furthermore, environmental factors such as sunlight and humidity play an important role in reducing bacterial loads on crop surfaces. For example, die-off rates of 0.52 log most-probable-number/day were observed for *E. coli* on lettuce under field conditions (42).

Alternative contamination sources often surpass manure in directly contributing to *E. coli* presence on crops. For instance, irrigation water contaminated with animal fecal matter has been identified as a major risk factor (43, 44). Similarly, wildlife activity in agricultural fields may introduce *E. coli*, as underlined by Merchant (20), especially in areas with high wildlife density. Greenhouse cultivation systems further complicate the issue, as such environments create conditions favorable for prolonged *E. coli* persistence (45). Avery et al. (17) highlighted the influence of environmental factors, such as rainfall, on the transfer of pathogens from manure to crops. *E. coli* from livestock feces could survive on grass for up to 6 months under conducive conditions, thereby increasing the risk of contamination through runoff or splash effects during heavy rainfall.

Research by Sun et al. demonstrated that the primary pathway for the transmission of manure-borne microbes and ARGs to lettuce is from surface soil to the leaf episphere. The study found that ~81% of the microbes and 62% of the ARGs present in the lettuce episphere originated from surface soil, pointing up the significant role of splashing during irrigation and direct contact in facilitating contamination from manure-amended soils to leafy greens. Notably, manure had limited effects on the rhizosphere microbiome and associated resistant genes. This suggests that horizontal transfer (the physical movement of manure-associated bacteria or free DNA, via splash or irrigation) from surface soil rather than uptake through roots, represents the dominant pathway for contamination of crops by manure-borne ARGs (46).

The effects of poultry litter soil amendments on *E. coli* and antibiotic-resistant strains have also been explored. For instance, an increase in tetracycline- and third-generation cephalosporin-resistant *E. coli* populations was observed during the first 28 days following application, with levels returning to baseline by day 70. These results suggest that while raw poultry litter may transiently enrich antibiotic-resistant *E. coli*, its long-term impact is minimal in managed cropping systems. Additionally, cover cropping has been shown to further reduce tetracycline-resistant *E. coli* levels, offering an effective supplemental mitigation strategy (47).

Manure management practices such as composting and adherence to pre-harvest intervals are essential for mitigating contamination risks (48). Manure-related *E. coli* contamination of crops depends on pathways such as surface splash, irrigation, and internalization via roots. While contamination risks are reduced by proper manure management and pre-harvest intervals, practices like raw manure application carries a *low to medium risk-ranking*, with increased probability when environmental conditions promote bacterial transfer.

## Water contamination

### Surface water

Manure application significantly contributes to *E. coli* contamination in surface waters through runoff during precipitation events and improper manure management. There are evidences that demonstrates that raindrop impact enhances *E. coli* detachment from manure-amended soils, leading to increased bacterial runoff into adjacent water bodies (3). Field experiments indicated that mitigating raindrop impacts, such as by maintaining vegetative cover, substantially reduced *E. coli* transport during runoff events (49). A study by Amato et al. observed that poultry litter applied to croplands contributed to increased presence of cephalosporin-resistant *E. coli* in streams within the Chesapeake Bay area in the USA. The study found that the density of poultry barns was positively correlated with increased resistance in *E. coli* between 6.2 and 18.9%, indicating that manure from these operations is a critical source of nutrient pollution and antibiotic-resistant bacteria in nearby water bodies (2). Alegbeleye et al. (50) and Jacobs et al. (51) further underscore that hydrological drivers such as storms and heavy rainfall significantly influence the transport of manure-borne pathogens into water bodies.

Cook (52) reported high genetic diversity of *E. coli* in agriculturally impacted streams, suggesting multiple contamination sources, including manure runoff and wildlife contributions. The role of poultry litter in surface water contamination is further underlined by Hubbard (53) who detected *E. coli* in streams near large-scale poultry operations. It was further highlighted that contaminated irrigation water can serve as a vector for *E. coli* transport, leading to internalization into lettuce tissues (6, 54).

### Groundwater

Groundwater contamination by *E. coli* is less frequent but poses significant risks in regions with porous soils and high manure application rates. *E. coli* contamination was detected in groundwater following livestock manure applications, even with a 12-meter-thick unsaturated zone comprising coarse and heterogeneous glacial sediments (55).

Further supporting this another study, investigated the leaching of *E. coli* and other pathogens through intact soil cores following surface application and injection of slurry. The study found that under natural weather conditions, microorganisms from manure could be transported through the soil into groundwater, posing a risk to water quality (56).

There are also indications that soil type, tillage practice, and the presence of manure influence the leaching of *E. coli* O157:H7, suggesting that different soil conditions and agricultural practices significantly impact the vertical movement of pathogens (57).

For manure to water systems, the contamination risk increases significantly, especially for surface waters and was assigned as *high environmental contamination risk-ranking*. Sowah and Hubbard highlight that manure runoff during rainfall events is a primary contributor to *E. coli* and resistant bacteria presence in streams, particularly near confined feeding operations. This contamination risk is exacerbated by poor manure storage and runoff controls (3, 53). Additionally, groundwater contamination, although categorized as a lower risk compared to surface waters, remains significant in regions with porous soils and high manure application rates (55, 58).

By identifying non-negligible pathways of *E. coli* transfer (Objective i), our map of soil, crop and water routes can serve as the structural backbone for QMRA risk pathways. Second, the quantitative persistence and concentration data compiled (Objective ii) provide the parameter values needed for the exposure assessment. Furthermore, the interventions we outline (Objective iii) such as composting, anaerobic digestion of manure, short-term poultry litter storage and pre-harvest intervals can be directly incorporated in QMRA as mitigation options to compare reduction in human exposure. Finally, by highlighting research gaps (Objective iv), we flag where the QMRA will be most uncertain and where future experiments should focus to increase model robustness.

## Limitations

This work was conducted as an exploratory narrative review and the number of studies screened, excluded and included in this work, was not systematically recorded. We opted for an exploratory because the available studies differed widely, making a single, unified protocol impossible. Furthermore, the literature selection and screening process was performed by a single person. The manure treatments section provides only a broad overview of the current state of research and does not analyse in depth all possible treatment methods. Although our search terms did not restrict by region, the preponderance of North American and European studies reflects where research has been published, not our methodology. We searched both PubMed and Google Scholar without geographical filters, so the observed bias stems from the literature itself.

## Research gaps

This review identifies several critical gaps in the literature on the environmental transfer of *E. coli* from manure:

- *Focus on resistant strains*: research predominantly examines general *E. coli*, with limited attention to antibiotic-resistant strains and their persistence.
- *Limited water contamination data*: studies on manure's impact on *E. coli* contamination of water systems, especially groundwater, are scarce compared to soil and crops research.
- *Heterogeneity in study design*: variability in experimental conditions, manure types, and methodologies complicates data synthesis and application to specific agricultural contexts.
- *Geographical bias*: research is predominantly concentrated in specific regions, with most studies originating from North America, followed by Europe. Other regions, such as Africa, Asia, South America, and Oceania, are significantly underrepresented. This imbalance underscores the need for more studies in these areas to ensure the global applicability of our findings and to address region-specific challenges.

## Conclusion

**Figure 1:**
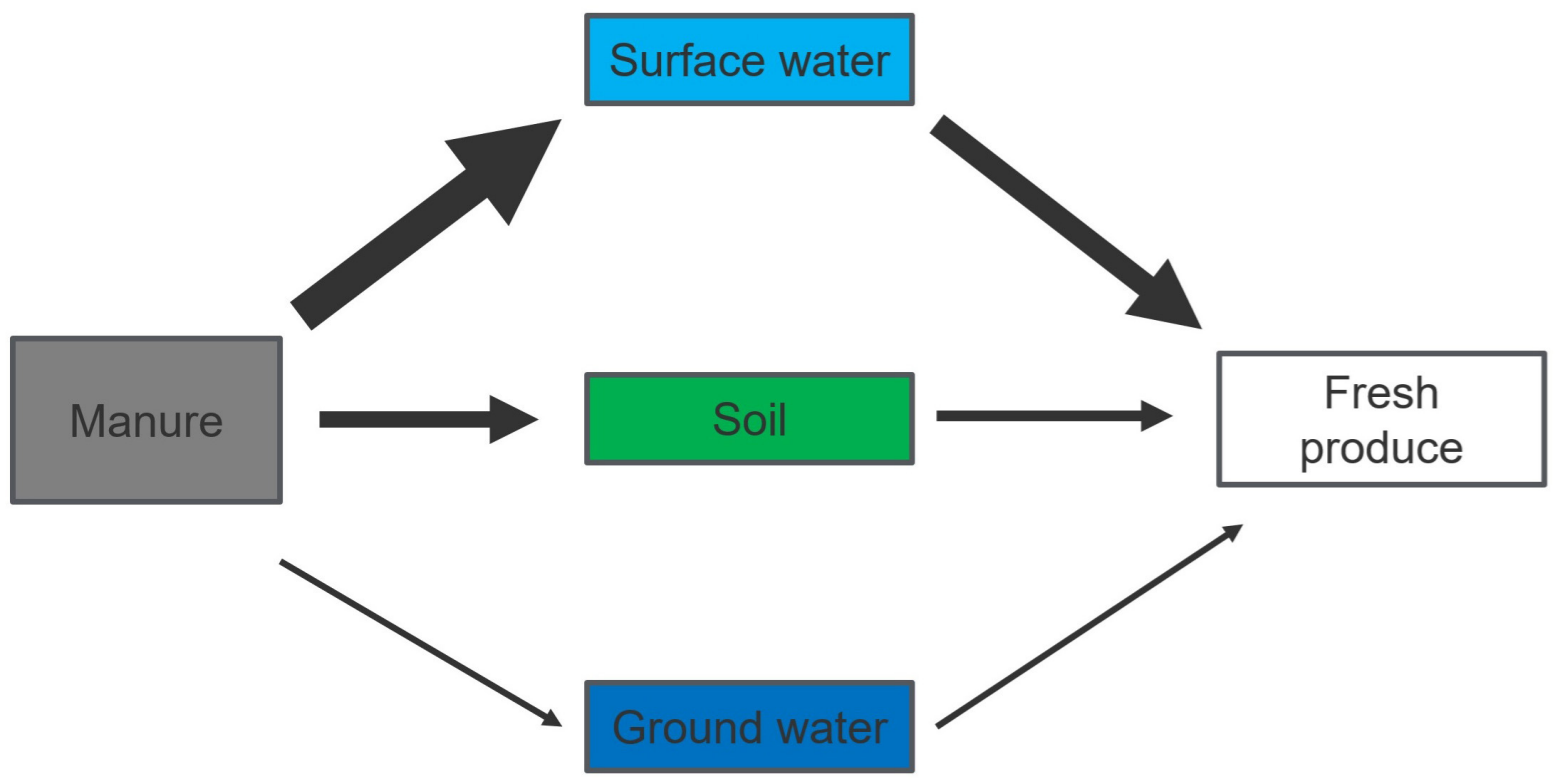
Relative contribution of selected risk pathways to *E. coli* human exposure via fresh produce consumption. Thicker arrow indicates higher risk, thinner arrow indicates lower risk or no evidences.

The transfer of *E. coli* from manure to soil, water, and crops poses significant environmental and public health challenge, with contamination risk depending on the pathway (Figure 1). Key findings include:

- *Soil contamination*: persistence of *E. coli* is influenced by manure type, treatment, and environmental factors with soil serving as a primary reservoir. Treated manure decreases the contamination risks but may not effectively reduce *E. coli* persistence length.
- *Crop contamination*: while it is possible for crops to be contaminated directly through manure application, other pathways, such as contaminated irrigation water and wildlife, may have a greater influence. These risks can be reduced substantially, however, through appropriate manure management and adherence to pre-harvest intervals.
- *Water contamination*: surface water contamination arises primarily from runoff during precipitation events. Groundwater contamination is less frequent but poses serious risks in vulnerable regions with porous soils.

There is a critical lack of studies on antibiotic-resistant *E. coli* and how long they survive in the environment compared to non-resistant strains. Future research should focus on standardized methodologies, regional challenges and interventions to address AMR risks in agriculture.
